# Practical Notes and Formulæ

**Published:** 1884-02-20

**Authors:** 


### PRACTICAL NOTES AND FORMULÆ.

**Suppositories for Piles.**—We translate the following recipe for suppositories for hæmorrhoids from the Zeitschrift fuer Therapie:

| | | | |
|---|---|---|---|
| ℞. | Iodoform | | 4 parts, |
| | Balsam Peru | | 8 " |
| | Cacao butter | of each | 5 " |
| | White wax | | |
| | Calcined magnesia | | 4 " |

Mix. To make twelve suppositories. One to be introduced after stool each time.—*Drug. Circular.*

**Formula for Hostetter's Bitters.**—

| | | |
|---|---|---|
| ℞ | Calamus root | 2 lbs., |
| | Orange peel | 2 " |
| | Peruvian bark | 2 " |
| | Gentian root | 2 " |
| | Colombo-root | 2 " |
| | Rhubarb | 8 ℥ |
| | Cinnamon | 4 ℥ |
| | Cloves | 2 ℥ |
| | Diluted alcohol | 4 gals., |
| | Water, pure | 2 " |
| | Sugar | 2 lbs. |

*New Remedies.*

**Eczema—Acute.**—

| | | |
|---|---|---|
| ℞. | Zinci carbonatis | aa. ʒ j, |
| | Plumbi carbonatis | |
| | Lac. sulphuris | |
| | Pulveris marantæ | |
| | Ungt. simplicis | ℥ j. |

Ft. ungt. molle.

Pencil lightly over the surface.—*Med. Summary.*

**For Asthma.**—The following is Dr. Fothergill's formula:

| | | |
|---|---|---|
| ℞. | Tinct. lobeliæ | ℥ v, |
| | Ammonii iodidi | ʒ ij, |
| | Ammonii bromidi | ʒ iij, |
| | Syr. totultani | ℥ iij. |

M. A teaspoonful every one, two, three or four hours. This gives relief in a few minutes, and sometimes the relief is permanent.—*Boston Jour. Chem.*

**Glycerite of Tar.**—The omission of this glycerite from the new Pharmacopœia renders desirable a preparation that may be made without difficulty, and will enable the pharmacist to furnish the various liquid preparations into which tar enters, in a cleanly, easy and expeditious manner. The glycerite made by the following formula being miscible with water in all proportions, and yielding a clear liquid, commends itself to the favorable consideration of pharmacists :

| | |
|---|---|
| Oil of tar | f. ℥ i, |
| Alcohol | f. ℥ ii, |
| Glycerin and water, each | f. ℥ iv, |
| Magnesium carbonate, q. s., or | ʒ vi. |

Mix the oil of tar with the alcohol, and rub these thoroughly with the magnesia to a smooth paste ; to this add the glycerin and water previously mixed together ; put the mixture into a well-corked bottle, and let it remain for several days, shaking it frequently ; then filter through paper.

For preparing syrup of tar add f. ℥ ii of glycerite to f. ℥ xiv of syrup.

Tar-water may be made very readily by using ℥ ss of glycerite and water sufficient to make a pint.

Wine of Tar may be made by using,

| | |
|---|---|
| Glycerite of tar | ℥ iii, |
| Sherry wine | ℥ iv, |
| Syrup | ℥ ii, |
| Water, enough to make | O i. |

Tar Ointment may be made much more easily and much smoother by using two drachms of oil of tar with six drahms of simple cerate.—*Journal of Pharmacy.*

**For Neuralgia.**—Let the patient take from three to five tablespoonfuls of the following mixture daily, either half an hour before the middle meal, or three hours after the same :

| | | |
|---|---|---|
| ℞. | Tinct. gelsemii | *m* c, |
| | Syrup simplic | fl. ʒ x, |
| | Aquæ destillat, q. s. ad. | ℥ viij. |

M This way the patient takes from fifteen to twenty-five drops of the tincture per day. It is not necessary to increase the dose still further.—*Med. and Surg. Rep.*

**Calomel as a Rat Poison.**—J. C., (Mt. Vernon, Ind.) writes as follows : "A correspondent in your December number, wished to know what could be added to paste to keep rats and mice from it. Let him put calomel in his paste, about one part in twenty, and the rats will most certainly let it alone. Calomel seems to act upon a rat as corrosive sublimate does upon man, and consequently is a sure death. I sell large quantities of it as rat poison.—*Druggists' Circular.*

**Needless, Useless Coughing.**—A favorite formula of Dr. Charles J. Hare, varied according to circumstances, is :

℞. Liq. morph. acetatis.......................... ʒ iij,

Acidi nitrici dil.......................... ʒ iss,

Oxymellis scillæ.......................... ʒ vj,

Mucil. acaciæ.......................... ℥ iiss,

Glycerini.......................... ʒ ij,

Syr. rhœados.......................... ℥ ij,

Aq. cinnam. (vel rosæ) ad.......................... ℥ vj.

M. To take one or two teaspoonfuls, five, six, or seven times in the twenty-four hours. The coughing in pertussis may be similarly relieved.—*Med. Age.*

**Asthma Mixture.**—

℞ Tinc. lobelia,.......................... } aa. ℥ ½,

Syr. of tolu,.......................... }

Camphor water.......................... ℥ 4.

M. Shake and give one tablespoonful to an adult every three hours.

**Itch Ointment.**—

℞. Naphthol.......................... 10 parts,
Cretæ albæ pulv.......................... 10 "
Saponis virdis.......................... 40 "
Adipis.......................... 40 "

—*New Remedies.*

**Cough Mixture.**—

℞. Syrup of tar.......................... 2 ounces,
Comp. syr. of squills.......................... 1 ounce.

M. A teaspoonful every three hours

**For Typhoid Fever.**—In typhoid fever, when the tongue is red and dry, give as a pleasant substitutef or turpentine, and at the same time possessing antiseptic and antiperodic properties, the fol lowing :

℞. Syrup of tar.......................... 4 ounces,
Fluid ext. eucalyptus.......................... ½ ounce.

M. Dose, one teaspoonful every four hours.

**How to Give Quinine to Children.**—Mix the dose in half a tablespoonful of the fluid extract of liquorice, and no bitterness will be noticed until a little while after the dose is swallowed, to guard against which give the child a stick of candy.

**A Pleasant Combination of Salicylate of Sodium.**—Editors Medical and Surgical Reporter: In No. 18 of present volume of your journal, "Country Doctor" wishes to know how to combine salicylate of sodium so as to make it pleasant. I send the following, also request him to look on page 19 of Stille & Maisch's Dispensatory, edition of 1879:

℞ Acid salicylic..........grs. c,
Borax..........grs. lxxx,
Syr. limonis..........℥ viii,
Aq. menth. pip..........℥ viii.
M.

**Syrup of Coffee.**—J. W. (Chicago, Ill.).—The following is recommended for use in administering quinine:

Finely ground coffee.......... 4 ounces,
Granulated sugar..........14 "
Boiling water, sufficient.

Exhaust the coffee by percolation with enough water to obtain eight fluid ounces of liquor, in which the sugar is to be dissolved at once without further heating. The coffee must be recently roasted, and should be ground immediately before the operation.—*Druggists' Circular and Chem. Gazette.*

**A Liniment for Rheumatism.**—The Quarterly Therapeutic Review says, methyl salicylate (oil of wintergreen) mixed with an equal quantity of olive oil, or linimentum saponis, applied externally to inflamed joints affected by acute rheumatism affords instant relief, and, having a pleasant odor, its use is very agreeable. —*Druggists' Circular.*

**For Irritable Bladder.**—

℞ Brom. potassium..........ʒ ij,
Tinc. gelsemium..........gtts. lx,
Water..........℥ iij.
M. Teaspoonful every two hours for a few days.

**Anodyne.**—

℞ Tinc. Lactucarium.......... ʒ iij,

Simple syrup..........℥ ss,

Camphor water..........℥ iv.

M. Dose, one tablespoonful every two to four hours. A good substitute for paregoric in children, for whom the dose must be reduced to suit the age.

**Aphrodisiac.**—Dr. Ellerslie Wallace describes nux vomica as the great invigorator of the sexual organs. He gives the one-half to one grain dose of the extract of nux vomica three times a day, after meals.—*Ex.*

